# Supplementary material for: Change in hierarchy of the financial networks: A study on firms of an emerging market in Bangladesh
Source: PLoS One. 2024 May 31;19(5):e0301725. doi: 10.1371/journal.pone.0301725 (PMC11142525; doi:10.1371/journal.pone.0301725)
Supplement: S1 Appendix — (DOCX) [file pone.0301725.s001.docx]

**Appendix**

**A. Full forms of abbreviations**

BWEL: Bangladesh Welding Electrodes Limited

CCL: Confidence Cement Limited

CGICL: City General Insurance Company Limited

EHL: Eastern Housing Limited

FICL: Federal Insurance Company Limited

FWFL: FU-WANG Foods Limited

GSL: Golden Son Limited

IFICB: International Finance Investment and Commerce Bank Limited

KCL: Keya Cosmetics Limited

LBFL: LankaBangla Finance Limited

MICL: Mercantile Insurance Company Limited

NCCBL: National Credit and Commerce Bank Limited

PBL: The Premier Bank Limited

RICL: Rupali Insurance Company Limited

SBIL: Sonar Bangla Insurance Limited

SBL: Standard Bank Limited

TBL: Trust Bank Limited

**B. Node-label to company name map**

| **Node label** | **Company name** | **Node label** | **Company name** |
| --- | --- | --- | --- |
| 0 | AB BANK LIMITED | 55 | LANKA-BANGLA FINANCE LTD. |
| 1 | ADVANCED CHEMICAL INDUSTRIES LIMITED | 56 | MEGHNA CEMENT MILLS LIMITED |
| 2 | AFTAB AUTOMOBILES LIMITED. | 57 | MEGHNA CONDENSED MILK INDUSTRIES LTD. |
| 3 | AGNI SYSTEMS LTD. | 58 | MEGHNA PETROLEUM LIMITED |
| 4 | AGRANI INSURANCE CO. LTD. | 59 | MERCANTILE BANK LIMITED |
| 5 | AGRICULTURAL MARKETING COMPANY LIMITED | 60 | MERCANTILE INSURANCE CO. LTD. |
| 6 | AL-ARAFAH ISLAMI BANK LTD. | 61 | METRO SPINNING LTD. |
| 7 | AMBEE PHARMACEUTICALS LIMITED | 62 | MIRACLE INDUSTRIES LIMITED. |
| 8 | APEX FOODS LIMITED | 63 | MITHUN KNITTING AND DYEING(CEPZ) LTD. |
| 9 | APEX TANNERY LIMITED | 64 | MONNO CERAMIC INDUSTRIES LTD. |
| 10 | ARAMIT CEMENT LTD. | 65 | MUTUAL TRUST BANK LTD. |
| 11 | ARAMIT LIMITED | 66 | NATIONAL BANK LIMITED. |
| 12 | ATLAS BANGLADESH LIMITED. | 67 | NATIONAL CREDIT AND COMMERCE BANK LIMITED |
| 13 | BANGLADESH EXPORT IMPORT COMPANY LIMITED | 68 | NATIONAL LIFE INSURANCE COMPANY LIMITED |
| 14 | BANGLADESH FINANCE AND INVESTMENT COMPANY LTD. | 69 | NATIONAL POLYMER INDUSTRIES LIMITED |
| 15 | BANGLADESH GENERAL INSURANCE COMPANY LIMITED | 70 | NATIONAL TUBES LIMITED. |
| 16 | BANGLADESH LAMPS LIMITED. | 71 | OLYMPIC INDUSTRIES LIMITED. |
| 17 | BANGLADESH THAI ALUMINIUM LIMITED. | 72 | ONE BANK LIMITED |
| 18 | BANGLADESH WELDING ELECTRODES LTD. | 73 | PARAMOUNT INSURANCE CO. LTD. |
| 19 | BANK ASIA LIMITED | 74 | PEOPLES INSURANCE COMPANY LIMITED |
| 20 | BATA SHOE COMPANY(BANGLADESH) LTD. | 75 | PHOENIX FINANCE AND INVESTMENT LTD. |
| 21 | BDCOM ONLINE LIMITED. | 76 | PHOENIX INSURANCE COMPANY LIMITED |
| 22 | BEXIMCO PHARMACEUTICALS LIMITED | 77 | PIONEER INSURANCE COMPANY LTD. |
| 23 | BRAC BANK LTD. | 78 | PRAGATI INSURANCE LIMITED |
| 24 | BRITISH AMERICAN TOBACCO BANGLADESH COMPANY LIMITED | 79 | PREMIER LEASING & FINANCE LTD. |
| 25 | CITY GENERAL INSURANCE CO. LTD. | 80 | PRIME BANK LTD. |
| 26 | CONFIDENCE CEMENT LIMITED | 81 | PRIME FINANCE & INVESTMENT LTD. |
| 27 | DELTA SPINNERS LTD. | 82 | PRIME INSURANCE COMPANY LTD. |
| 28 | DHAKA BANK LIMITED. | 83 | PRIME ISLAMI LIFE INSURANCE LTD. |
| 29 | DUTCH BANGLA BANK LTD. | 84 | PRIME TEXTILE SPINNING MILLS LTD. |
| 30 | DHAKA ELECTRIC SUPPLY COMPANY LTD. | 85 | PUBALI BANK LIMITD. |
| 31 | EASTERN BANK LIMITED. | 86 | POWER GRID COMPANY OF BANGLADESH LTD. |
| 32 | EASTERN HOUSING LIMITED | 87 | RANGPUR FOUNDRY LTD. |
| 33 | EASTERN INSURANCE COMPANY LIMITED | 88 | RENATA LIMITED |
| 34 | EASTLAND INSURANCE COMPANY LIMITED | 89 | RUPALI INSURANCE COMPANY LIMITED |
| 35 | EXPORT IMPORT BANK OF BD. LTD. | 90 | S. ALAM COLD ROLLED STEELS LTD. |
| 36 | FAREAST ISLAMI LIFE INSURANCE CO. | 91 | SAIHAM TEXTILE MILLS LTD. |
| 37 | FEDERAL INSURANCE COMPANY LIMITED | 92 | SANDHANI LIFE INSURANCE COMPANY LIMITED |
| 38 | FINE FOODS LIMITED | 93 | SHAHJALAL ISLAMI BANK LTD. |
| 39 | FU-WANG CERAMIC INDUSTRY LIMITED | 94 | SINGER BANGLADESH LIMITED. |
| 40 | FU-WANG FOODS LIMITED | 95 | SINOBANGLA INDUSTRIES LTD. |
| 41 | GLOBAL INSURANCE LTD. | 96 | SONAR BANGLA INSURANCE LTD. |
| 42 | GQ BALL PEN INDUSTRIES LIMITED | 97 | SOUTHEAST BANK LIMITED. |
| 43 | GOLDEN SON LTD. | 98 | SQUARE PHARMACEUTICALS LIMITED |
| 44 | H.R. TEXTILE MILLS LIMITED | 99 | SQUARE TEXTILES LTD. |
| 45 | INFORMATION SERVICES NETWORK LTD. | 100 | STANDARD BANK LIMITED |
| 46 | INTERNATIONAL FINANCE INVESTMENT AND COMMERCE BANK LIMITED | 101 | SUMMIT POWER LTD. |
| 47 | INTERNATIONAL LEASING & FINANCIAL SERVICES LTD. | 102 | THE CITY BANK LIMITED. |
| 48 | INVESTMENT CORPORATION OF BANGLADESH. | 103 | THE IBN SINA PHARMACEUTICAL INDUSTRY LTD. |
| 49 | ISLAMI BANK BANGLADESH LIMITED. | 104 | THE PREMIER BANK LTD. |
| 50 | ISLAMIC FINANCE & INVESTMENT LTD. | 105 | TRUST BANK LIMITED |
| 51 | JAMUNA OIL COMPANY LIMITED | 106 | USMANIA GLASS SHEET FACTORY LIMITED |
| 52 | JAMUNA BANK LTD. | 107 | UTTARA BANK LIMITED. |
| 53 | KARNAPHULI INSURANCE COMPANY LIMITED | 108 | UTTARA FINANCE AND INVESTMENT COMPANY LIMITED |
| 54 | KEYA COSMETICS LTD. | 109 | UNION CAPITAL LIMITED |
