# Supplementary material for: Change in hierarchy of the financial networks: A study on firms of an emerging market in Bangladesh
Source: PLoS One. 2024 May 31;19(5):e0301725. doi: 10.1371/journal.pone.0301725 (PMC11142525; doi:10.1371/journal.pone.0301725)
Supplement: S2 Appendix — (DOCX) [file pone.0301725.s002.docx]

**
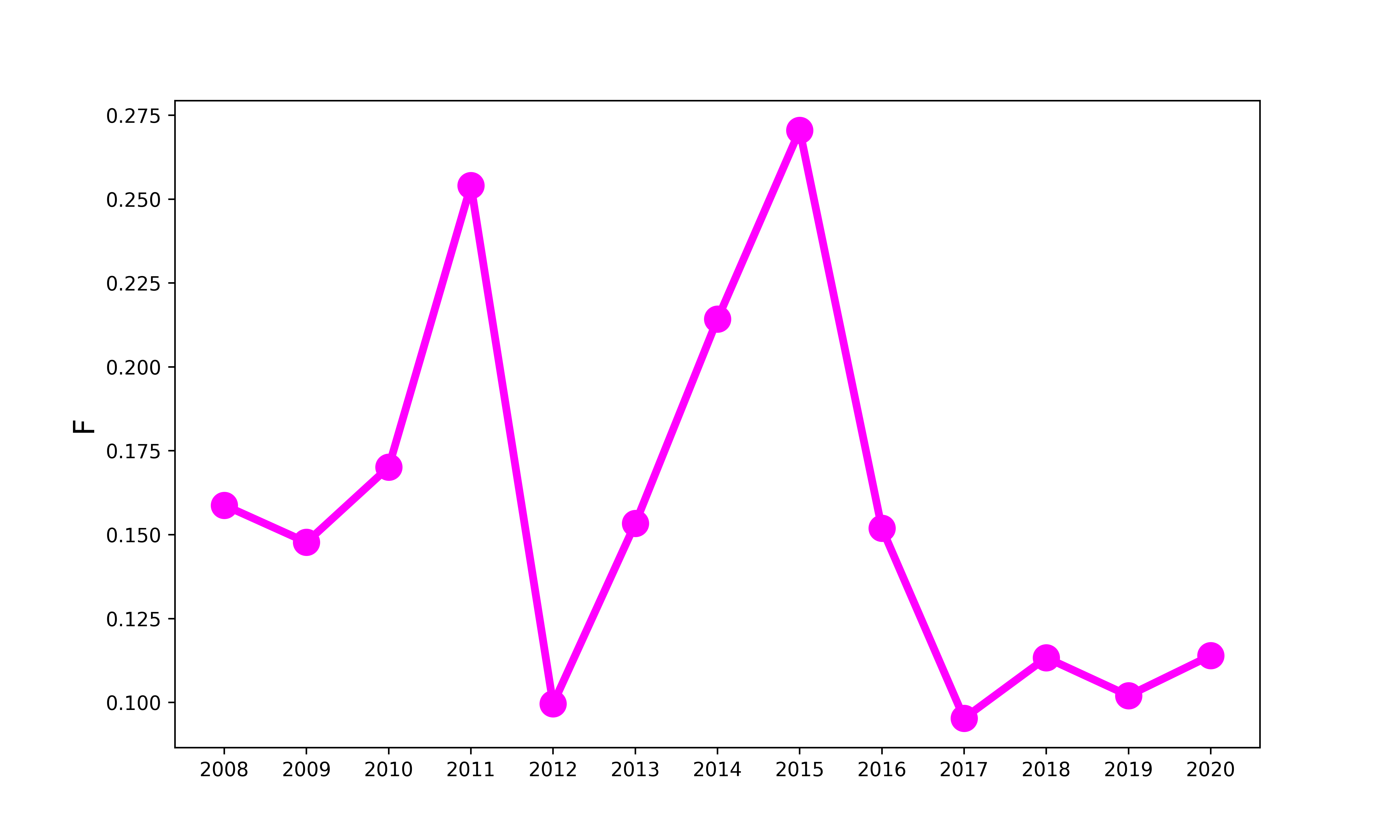
**

**Fig S4. Change of the hierarchy with the evolution of time for MST of S&P 500.**

**
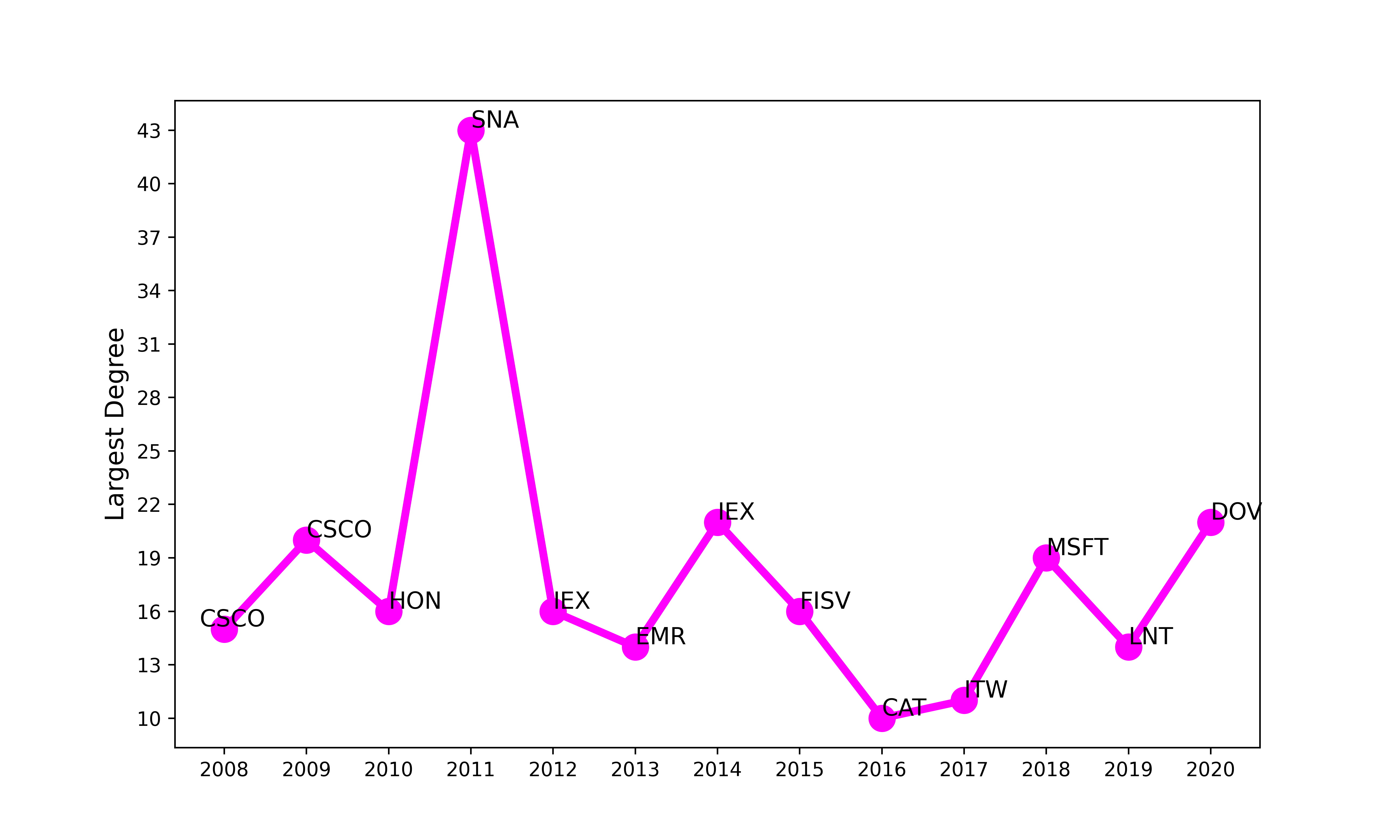
**

**Fig S5. Year-wise largest degree of stock companies in the S&P500 network.** The full names of the companies can be found in Table T3.


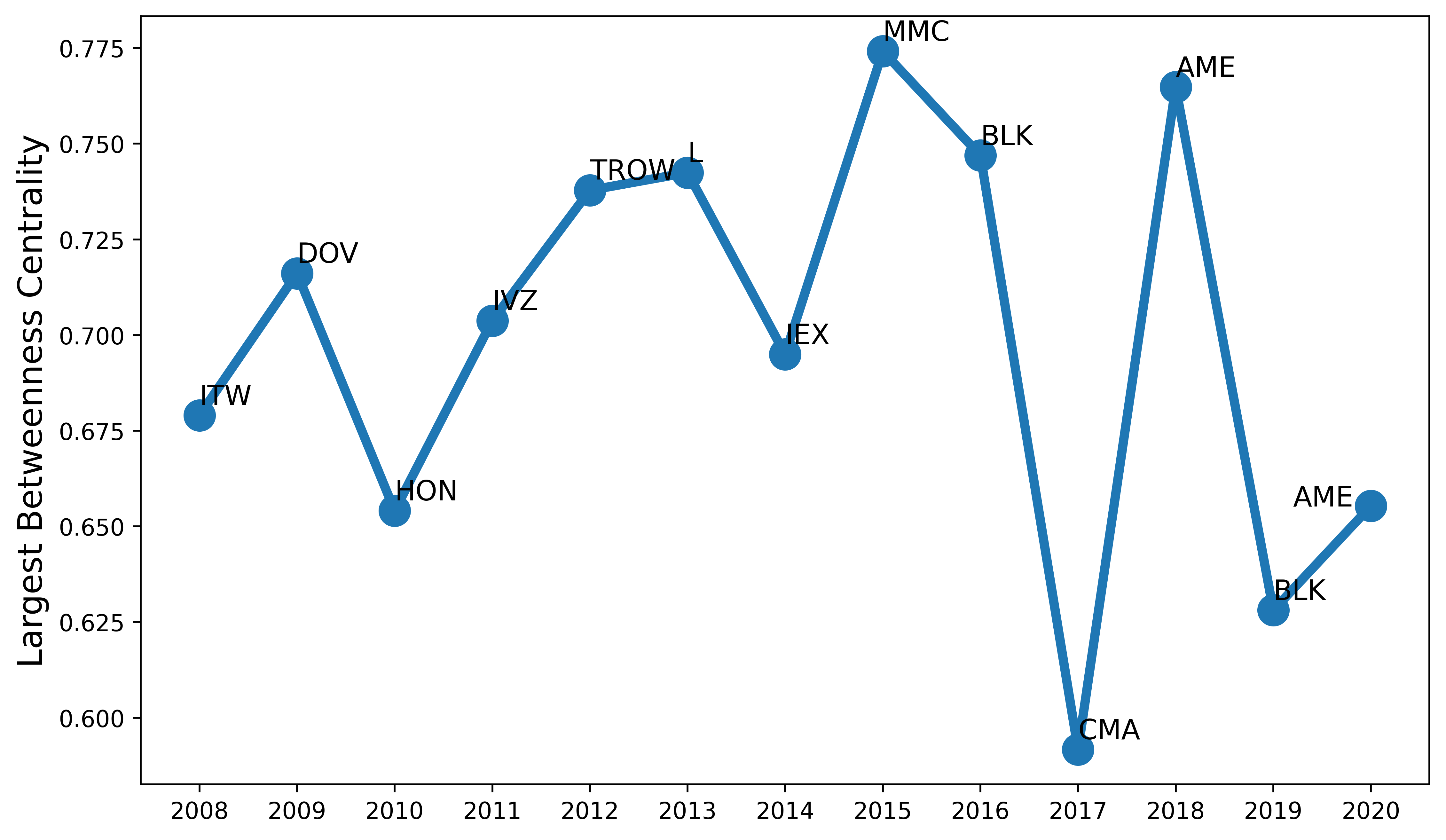


**Fig S6. Annual most betweenness central stock companies in the S&P500 network.** The full names of the companies can be found in Table T4.


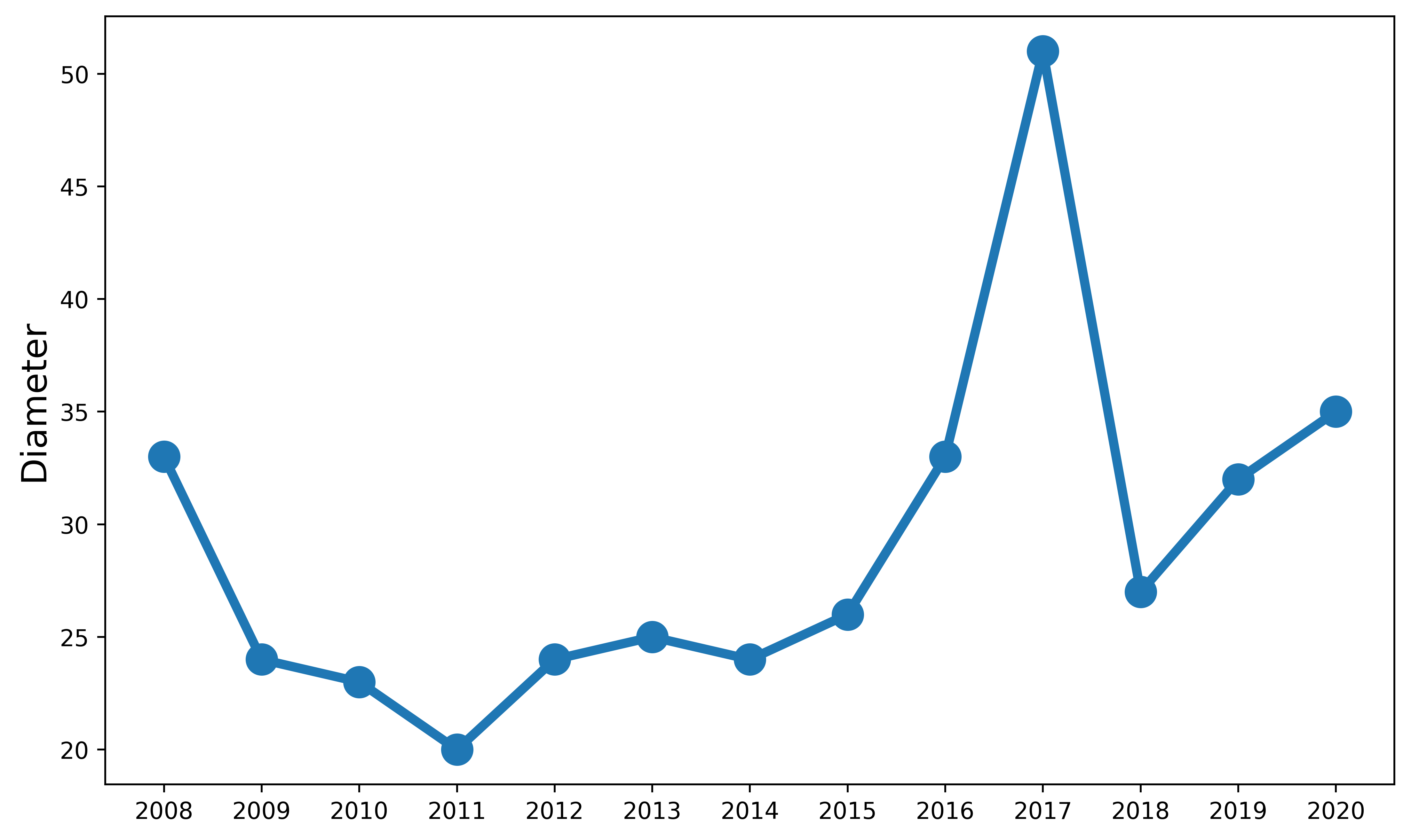

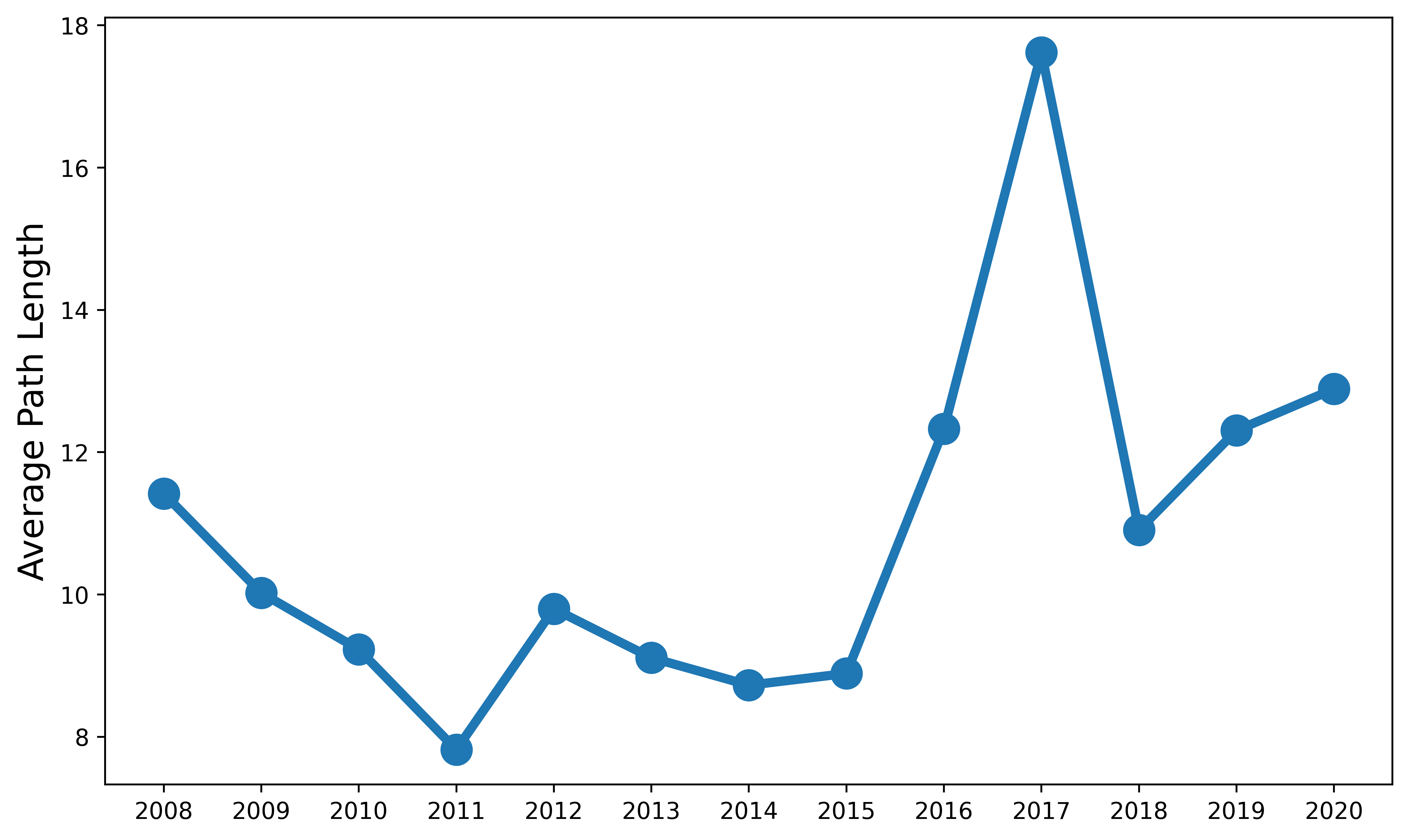


1. (b)

**Fig S7. Topological properties regarding the shortest path of the S&P500 network.** (a) Network Diameter (b) Average Shortest Path Length.

**Table T1: Hub ranking of nodes in the year 2008:**

| **Company Name** | **Degree** |
| --- | --- |
| NATIONAL CREDIT AND COMMERCE BANK LIMITED | 8 |
| BRAC BANK LTD. | 6 |
| MERCANTILE INSURANCE CO. LTD. | 6 |
| APEX TANNERY LIMITED | 5 |
| EASTLAND INSURANCE COMPANY LIMITED | 5 |
| APEX FOODS LIMITED | 4 |
| BANGLADESH LAMPS LIMITED. | 4 |
| BDCOM ONLINE LIMITED. | 4 |
| Golden Son Ltd. | 4 |
| LankaBangla Finance Ltd. | 4 |
| PHOENIX FINANCE AND INVESTMENT LTD. | 4 |
| PRIME TEXTILE SPINNING MILLS LTD. | 4 |
| Power Grid Company of Bangladesh Ltd. | 4 |
| RANGPUR FOUNDRY LTD. | 4 |
| AMBEE PHARMACEUTICALS LIMITED | 3 |
| BATA SHOE COMPANY(BANGLADESH) LTD. | 3 |
| BEXIMCO PHARMACEUTICALS LIMITED | 3 |
| FU-WANG FOODS LIMITED | 3 |
| GLOBAL INSURANCE LTD. | 3 |
| KARNAPHULI INSURANCE COMPANY LIMITED | 3 |
| MIRACLE INDUSTRIES LIMITED. | 3 |
| NATIONAL BANK LIMITED. | 3 |
| NATIONAL LIFE INSURANCE COMPANY LIMITED | 3 |
| ONE BANK LIMITED | 3 |
| PRIME INSURANCE COMPANY LTD. | 3 |
| STANDARD BANK LIMITED | 3 |
| The Premier Bank Ltd. | 3 |
| USMANIA GLASS SHEET FACTORY LIMITED | 3 |
| Union Capital Limited | 3 |
| AFTAB AUTOMOBILES LIMITED. | 2 |
| AGRICULTURAL MARKETING COMPANY LIMITED | 2 |
| ARAMIT LIMITED | 2 |
| BANGLADESH GENERAL INSURANCE COMPANY LIMITED | 2 |
| DELTA SPINNERS LTD. | 2 |
| EASTERN BANK LIMITED. | 2 |
| EASTERN HOUSING LIMITED | 2 |
| EASTERN INSURANCE COMPANY LIMITED | 2 |
| FU-WANG CERAMIC INDUSTRY LIMITED | 2 |
| H.R. TEXTILE MILLS LIMITED | 2 |
| INTERNATIONAL LEASING & FINANCIAL SERVICES LTD. | 2 |
| ISLAMIC FINANCE & INVESTMENT LTD. | 2 |
| Jamuna Bank Ltd. | 2 |
| MEGHNA CEMENT MILLS LIMITED | 2 |
| MEGHNA CONDENSED MILK INDUSTRIES LTD. | 2 |
| MEGHNA PETROLEUM LIMITED | 2 |
| METRO SPINNING LTD. | 2 |
| OLYMPIC INDUSTRIES LIMITED. | 2 |
| PARAMOUNT INSURANCE CO. LTD. | 2 |
| PREMIER LEASING & FINANCE LTD. | 2 |
| PRIME FINANCE & INVESTMENT LTD. | 2 |
| PRIME ISLAMI LIFE INSURANCE LTD. | 2 |
| S. Alam Cold Rolled Steels Ltd. | 2 |
| SANDHANI LIFE INSURANCE COMPANY LIMITED | 2 |
| SQUARE TEXTILES LTD. | 2 |
| THE CITY BANK LIMITED. | 2 |
| AB BANK LIMITED | 1 |
| ADVANCED CHEMICAL INDUSTRIES LIMITED | 1 |
| AGNI SYSTEMS LTD. | 1 |
| AGRANI INSURANCE CO. LTD. | 1 |
| AL-ARAFAH ISLAMI BANK LTD. | 1 |
| ARAMIT CEMENT LTD. | 1 |
| ATLAS BANGLADESH LIMITED. | 1 |
| BANGLADESH EXPORT IMPORT COMPANY LIMITED | 1 |
| BANGLADESH FINANCE AND INVESTMENT COMPANY LTD. | 1 |
| BANGLADESH THAI ALUMINIUM LIMITED. | 1 |
| BANGLADESH WELDING ELECTRODES LTD. | 1 |
| BANK ASIA LIMITED | 1 |
| BRITISH AMERICAN TOBACCO BANGLADESH COMPANY LIMITED | 1 |
| CITY GENERAL INSURANCE CO. LTD. | 1 |
| CONFIDENCE CEMENT LIMITED | 1 |
| DHAKA BANK LIMITED. | 1 |
| DUTCH BANGLA BANK LTD. | 1 |
| Dhaka Electric Supply Company Ltd. | 1 |
| EXPORT IMPORT BANK OF BD. LTD. | 1 |
| FAREAST ISLAMI LIFE INSURANCE CO. | 1 |
| FEDERAL INSURANCE COMPANY LIMITED | 1 |
| FINE FOODS LIMITED | 1 |
| GQ BALL PEN INDUSTRIES LIMITED | 1 |
| INFORMATION SERVICES NETWORK LTD. | 1 |
| INTERNATIONAL FINANCE INVESTMENT AND COMMERCE BANK LIMITED | 1 |
| INVESTMENT CORPORATION OF BANGLADESH. | 1 |
| ISLAMI BANK BANGLADESH LIMITED. | 1 |
| JAMUNA OIL COMPANY LIMITED | 1 |
| KEYA COSMETICS LTD. | 1 |
| MERCANTILE BANK LIMITED | 1 |
| MITHUN KNITTING AND DYEING(CEPZ) LTD. | 1 |
| MONNO CERAMIC INDUSTRIES LTD. | 1 |
| MUTUAL TRUST BANK LTD. | 1 |
| NATIONAL POLYMER INDUSTRIES LIMITED | 1 |
| NATIONAL TUBES LIMITED. | 1 |
| PEOPLES INSURANCE COMPANY LIMITED | 1 |
| PHOENIX INSURANCE COMPANY LIMITED | 1 |
| PIONEER INSURANCE COMPANY LTD. | 1 |
| PRAGATI INSURANCE LIMITED | 1 |
| PRIME BANK LTD. | 1 |
| PUBALI BANK LIMITD. | 1 |
| RENATA LIMITED | 1 |
| RUPALI INSURANCE COMPANY LIMITED | 1 |
| SAIHAM TEXTILE MILLS LTD. | 1 |
| SHAHJALAL ISLAMI BANK LTD. | 1 |
| SINGER BANGLADESH LIMITED. | 1 |
| SINOBANGLA INDUSTRIES LTD. | 1 |
| SONAR BANGLA INSURANCE LTD. | 1 |
| SOUTHEAST BANK LIMITED. | 1 |
| SQUARE PHARMACEUTICALS LIMITED | 1 |
| SUMMIT POWER LTD. | 1 |
| THE IBN SINA PHARMACEUTICAL INDUSTRY LTD. | 1 |
| Trust Bank Limited | 1 |
| UTTARA BANK LIMITED. | 1 |
| UTTARA FINANCE AND INVESTMENT COMPANY LIMITED | 1 |

**Table T2: Hub ranking of nodes in the year 2020:**

| **Company Name** | **Degree** |
| --- | --- |
| The Premier Bank Ltd. | 11 |
| RANGPUR FOUNDRY LTD. | 8 |
| FEDERAL INSURANCE COMPANY LIMITED | 7 |
| APEX FOODS LIMITED | 6 |
| EASTERN HOUSING LIMITED | 6 |
| AGRANI INSURANCE CO. LTD. | 4 |
| FAREAST ISLAMI LIFE INSURANCE CO. | 4 |
| LankaBangla Finance Ltd. | 4 |
| MITHUN KNITTING AND DYEING(CEPZ) LTD. | 4 |
| NATIONAL POLYMER INDUSTRIES LIMITED | 4 |
| ONE BANK LIMITED | 4 |
| RUPALI INSURANCE COMPANY LIMITED | 4 |
| S. Alam Cold Rolled Steels Ltd. | 4 |
| AFTAB AUTOMOBILES LIMITED. | 3 |
| APEX TANNERY LIMITED | 3 |
| DUTCH BANGLA BANK LTD. | 3 |
| EASTLAND INSURANCE COMPANY LIMITED | 3 |
| FU-WANG CERAMIC INDUSTRY LIMITED | 3 |
| Golden Son Ltd. | 3 |
| INTERNATIONAL FINANCE INVESTMENT AND COMMERCE BANK LIMITED | 3 |
| ISLAMIC FINANCE & INVESTMENT LTD. | 3 |
| Jamuna Bank Ltd. | 3 |
| MEGHNA CEMENT MILLS LIMITED | 3 |
| MERCANTILE BANK LIMITED | 3 |
| NATIONAL TUBES LIMITED. | 3 |
| Power Grid Company of Bangladesh Ltd. | 3 |
| SINOBANGLA INDUSTRIES LTD. | 3 |
| THE CITY BANK LIMITED. | 3 |
| Union Capital Limited | 3 |
| AGNI SYSTEMS LTD. | 2 |
| BANGLADESH EXPORT IMPORT COMPANY LIMITED | 2 |
| BANGLADESH FINANCE AND INVESTMENT COMPANY LTD. | 2 |
| BATA SHOE COMPANY(BANGLADESH) LTD. | 2 |
| BEXIMCO PHARMACEUTICALS LIMITED | 2 |
| BRITISH AMERICAN TOBACCO BANGLADESH COMPANY LIMITED | 2 |
| EASTERN BANK LIMITED. | 2 |
| FU-WANG FOODS LIMITED | 2 |
| H.R. TEXTILE MILLS LIMITED | 2 |
| ISLAMI BANK BANGLADESH LIMITED. | 2 |
| JAMUNA OIL COMPANY LIMITED | 2 |
| KARNAPHULI INSURANCE COMPANY LIMITED | 2 |
| SAIHAM TEXTILE MILLS LTD. | 2 |
| SANDHANI LIFE INSURANCE COMPANY LIMITED | 2 |
| SONAR BANGLA INSURANCE LTD. | 2 |
| SOUTHEAST BANK LIMITED. | 2 |
| USMANIA GLASS SHEET FACTORY LIMITED | 2 |
| UTTARA BANK LIMITED. | 2 |
| UTTARA FINANCE AND INVESTMENT COMPANY LIMITED | 2 |
| AB BANK LIMITED | 1 |
| ADVANCED CHEMICAL INDUSTRIES LIMITED | 1 |
| AGRICULTURAL MARKETING COMPANY LIMITED | 1 |
| AL-ARAFAH ISLAMI BANK LTD. | 1 |
| AMBEE PHARMACEUTICALS LIMITED | 1 |
| ARAMIT CEMENT LTD. | 1 |
| ARAMIT LIMITED | 1 |
| ATLAS BANGLADESH LIMITED. | 1 |
| BANGLADESH GENERAL INSURANCE COMPANY LIMITED | 1 |
| BANGLADESH LAMPS LIMITED. | 1 |
| BANGLADESH THAI ALUMINIUM LIMITED. | 1 |
| BANGLADESH WELDING ELECTRODES LTD. | 1 |
| BANK ASIA LIMITED | 1 |
| BDCOM ONLINE LIMITED. | 1 |
| BRAC BANK LTD. | 1 |
| CITY GENERAL INSURANCE CO. LTD. | 1 |
| CONFIDENCE CEMENT LIMITED | 1 |
| DELTA SPINNERS LTD. | 1 |
| DHAKA BANK LIMITED. | 1 |
| Dhaka Electric Supply Company Ltd. | 1 |
| EASTERN INSURANCE COMPANY LIMITED | 1 |
| EXPORT IMPORT BANK OF BD. LTD. | 1 |
| FINE FOODS LIMITED | 1 |
| GLOBAL INSURANCE LTD. | 1 |
| GQ BALL PEN INDUSTRIES LIMITED | 1 |
| INFORMATION SERVICES NETWORK LTD. | 1 |
| INTERNATIONAL LEASING & FINANCIAL SERVICES LTD. | 1 |
| INVESTMENT CORPORATION OF BANGLADESH. | 1 |
| KEYA COSMETICS LTD. | 1 |
| MEGHNA CONDENSED MILK INDUSTRIES LTD. | 1 |
| MEGHNA PETROLEUM LIMITED | 1 |
| MERCANTILE INSURANCE CO. LTD. | 1 |
| METRO SPINNING LTD. | 1 |
| MIRACLE INDUSTRIES LIMITED. | 1 |
| MONNO CERAMIC INDUSTRIES LTD. | 1 |
| MUTUAL TRUST BANK LTD. | 1 |
| NATIONAL BANK LIMITED. | 1 |
| NATIONAL CREDIT AND COMMERCE BANK LIMITED | 1 |
| NATIONAL LIFE INSURANCE COMPANY LIMITED | 1 |
| OLYMPIC INDUSTRIES LIMITED. | 1 |
| PARAMOUNT INSURANCE CO. LTD. | 1 |
| PEOPLES INSURANCE COMPANY LIMITED | 1 |
| PHOENIX FINANCE AND INVESTMENT LTD. | 1 |
| PHOENIX INSURANCE COMPANY LIMITED | 1 |
| PIONEER INSURANCE COMPANY LTD. | 1 |
| PRAGATI INSURANCE LIMITED | 1 |
| PREMIER LEASING & FINANCE LTD. | 1 |
| PRIME BANK LTD. | 1 |
| PRIME FINANCE & INVESTMENT LTD. | 1 |
| PRIME INSURANCE COMPANY LTD. | 1 |
| PRIME ISLAMI LIFE INSURANCE LTD. | 1 |
| PRIME TEXTILE SPINNING MILLS LTD. | 1 |
| PUBALI BANK LIMITD. | 1 |
| RENATA LIMITED | 1 |
| SHAHJALAL ISLAMI BANK LTD. | 1 |
| SINGER BANGLADESH LIMITED. | 1 |
| SQUARE PHARMACEUTICALS LIMITED | 1 |
| SQUARE TEXTILES LTD. | 1 |
| STANDARD BANK LIMITED | 1 |
| SUMMIT POWER LTD. | 1 |
| THE IBN SINA PHARMACEUTICAL INDUSTRY LTD. | 1 |
| Trust Bank Limited | 1 |

**Table T3:**

| **Company Symbol** | **Company Name** | **Sector** |
| --- | --- | --- |
| CSCO | Cisco | Information Technology |
| HON | Honeywell | Industrials |
| SNA | Snap-on | Industrials |
| IEX | IDEX Corporation | Industrials |
| EMR | Emerson Electric | Industrials |
| FISV | Fiserv, Inc. | Financials |
| CAT | Caterpillar Inc. | Industrials |
| ITW | Illinois Tool Works | Industrials |
| MSFT | Microsoft | Information Technology |
| LNT | Alliant Energy | Utilities |
| DOV | Dover Corporation | Industrials |

**Table T4:**

| **Company Symbol** | **Company Name** | **Sector** |
| --- | --- | --- |
| IVZ | Invesco | Financials |
| TROW | T. Rowe Price | Financials |
| L | Loews Corporation | Financials |
| ITW | Illinois Tool Works | Industrials |
| DOV | Dover Corporation | Industrials |
| HON | Honeywell | Industrials |
| MMC | Marsh McLennan | Financials |
| BLK | BlackRock | Financials |
| CMA | Comerica | Financials |
| IEX | IDEX Corporation | Industrials |
| AME | Ametek | Industrials |
