# Supplementary material for: Change in hierarchy of the financial networks: A study on firms of an emerging market in Bangladesh
Source: PLoS One. 2024 May 31;19(5):e0301725. doi: 10.1371/journal.pone.0301725 (PMC11142525; doi:10.1371/journal.pone.0301725)
Supplement: Attachment — Submitted filename: Response to Reviewers.docx [file pone.0301725.s003.docx]

**Rebuttal letter**

PONE-D-23-35741

Change in hierarchy of the financial networks: A study on firms of an emerging market in Bangladesh

PLOS ONE

10 February 2024

We thank the editor and the reviewers for their valuable comments on our manuscript. Below is our response to each point raised by the academic editor and reviewers. We hope that we satisfyingly addressed them and that the manuscript will be now suited for publication.

We edit our manuscript according to the requisitions of the editor and reviewers. The modifications are inserted in red font in the “Revised Manuscript with Track Changes” file. However, some changes could not be marked properly as we had to reorganize the whole manuscript and eliminate redundancies while addressing the editor’s and reviewers’ comments.

Sincerely,

On behalf of all authors,

Dr. Ashadun Nobi

**Responds to Academic Editor:**

**Query 1:** It seems that either formula (2) is incorrect or has been entered incorrectly.

**Response:** Formula (2) is corrected. Thank you.

**Query 2:** A chart for the DSE stock index for the same period should be presented in Fig. 1 or separately.

**Response:** The DSE stock index chart for the same period is inserted in Fig. 1(b).

**Query 3:** It is hard to agree with the presented conclusions on page 17 (lines 337-380). The COVID-19 pandemic originated in December 2019 in China. Did the financial market in Bangladesh anticipate the negative impact of COVID-19 on its functioning? Or is it, however, the result of other macroeconomic factors, including systematic risk? The COVID-19 pandemic is considered a Black Swan event in the financial market, a completely unpredictable occurrence with broad implications for financial markets. In my opinion, this passage should be reformulated, attempting to find alternative explanations for changes in the Hierarchy coefficient.

**Response:** We agree with you that the COVID-19 pandemic is considered a Black Swan event, which is unpredictable from the financial perspective. Following this, we provide an alternative explanation of the relationship between the COVID-19 pandemic and hierarchical change. We have said that the hierarchy fell sharply in 2019 and 2020, implying that the market is in turbulence and that a financial shock is on the way may be as a result of the Black Swan COVID-19 or some other macroeconomic factors. We have removed those lines that might lead to a wrong interpretation of the COVID-19 prediction based on financial context.

**Responds to Reviewers:**

**Reviewer 1:**

**Query 1:** What are your contributions? Better highlight novelty in the study. Better define motivations for the research.

**Response:** The aim of this study is to analyse the time series of an emerging market from 2008 to 2020, covering different crises using a statistical and complex network approach and to compare the hierarchical structure of this market with other emergent, developing and developed market done in last decades. The contribution of our study is that we investigate the hierarchical structure and organization of an emerging financial market, e.g., the DSE of Bangladesh in this research, for the first time, which might help the authorities and policymakers of this and similar markets to identify behavioural differences from that of developed and developing markets that can be used in promoting them to the next higher levels. We showed the significant differences in the hierarchical structure of the emergent market's tree during different crises than those of developed and developing markets not seen before. In the manuscript, we have added a detailed explanation of the motivation of our research.

**Query 2:** Page 3, line 14. As far as I know, the Chinese stock market is still an emerging market compared to the U.S. Please ensure the expression is appropriate.

**Response:** We corrected it in the manuscript.

**Query 3:** Introduction - Lack of appropriate critical appraisal of included studies to find current research gaps.

**Response:** We have made significant efforts to organize the introduction part and have included additional points to enhance the clarity of the research gap.

**Query 4:** The choice of MST needs to be motivated. The authors merely list alternative models, such as random matrix theory, wavelet method, principal component analysis, and GARCH method. Instead, they should present a comprehensive summary of the advantages and disadvantages of each method.

**Response:** We just mentioned the alternative models since they were previously used to analyse financial time series data. However, our aim is to analyse the hierarchical structure of a financial network. We explore the hierarchical technique in depth, which is considerably different from above methods.

**Query 5:** PMFG preserves the same hierarchical structure as MST but offers more significant and richer information. Thus, the question arises: Why would one choose to utilize MST over PMFG.

**Response:** For the purpose of our research, we measured the network's hierarchy using hierarchical path. The calculation of hierarchical path in MST is much easier due to the lack of cycles and lower number of edges. Conversely, PMFG can contain cycles and it has higher number of edges which makes number of paths between two nodes exponentially large and hierarchy computation becomes almost impossible. That’s why we used MST in our research.

**Query 6:** The last waves of COVID-19 and the Russia-Ukraine conflict are two essential events for financial stability. The authors should cover these periods.

**Response:** The Bangladesh Securities and Exchange Commission (BSEC) imposed the floor price on all listed shares in the Dhaka Stock Exchange (DSE) in June 2022 to protect small investor’s capital. The floor price was set to prevent the share prices from falling below a certain level, which would have caused significant losses for small investors [1][2]. The imposition of the floor price in the DSE will hinder our ability to obtain accurate information regarding the effects of the Russia-Ukraine war on the DSE.

**Query 7:** The DSE, such abbreviations, must be defined at their first mention there.

**Response:** We fixed it in the manuscript.

**Query 8:** Line 289. The hub and the peripheral are measured by which of the centralities? The author should also show the hub ranking of all assets.

**Response:** We identified nodes as hub and peripheral based on the extent of their connectivity. The node with the highest degree in the tree is referred to as the hub node, while the nodes with the lower degree that lie outskirt in the tree are termed as peripheral nodes. We added this information in the manuscript and we provided the hub ranking of all assets in the supplementary document S2 Appendix (Table T1 and Table T2).

**Query 9:** In Fig.1. The author should elaborate on why the peripheral company, AB Bank Limited, fell sharply in 2012, and Advance Chemical Industries Limited had a different trend compared to the other three companies during 2010 and 2011.

**Response:** Before the financial market crash of 2010–2011, most of the company's prices were overestimated than regular prices, the reason behind this is unknown and we leave it for further investigation. The companies whose prices are not overestimated, like AB Bank Limited and Advanced Chemical Industries Limited (ACI), did not go down during the crisis of 2010-2011. Even though AB Bank stayed out of the financial crisis in 2011, the company's price dropped in 2012 because of loan fraud and the impact of a sponsor director. ACI's pricing remained consistent and distinct from that of other companies in the DSE due to its diverse business operations across multiple sectors, good budgeting, cost control, and strategic financial planning. We added this information in the manuscript.

**Query 10:** Figs. 4-7. The authors should supplement the U.S. market in figures as a contrast.

**Response:** U.S. market figures are added in the supplementary document.

**Query 11:** The conclusion section seems to rush to the end. The authors will have to demonstrate the impact and insights of the research. Clearly state your unique research contributions in the conclusion section.

**Response:** Conclusion section is modified in the manuscript according to the query.

**Reviewer 2:**

**Query 1:** The authors should restructure the article, avoiding redundancies and focusing more on results and analyses. The article contains a lot of information that obscures the central theme of the research.

**Response:** Following your comment, we have eliminated redundancies from different sections of the manuscript, which has shortened the article size. Besides, some less important information has also been removed to focus more on the main theme of the paper.

**Query 2:** Some statistical analyses deserve more attention. For me, it is not surprising that the distribution of sample correlations deviates from the Gaussian distribution.

**Response:** In this article, we provide statistical analyses to relate the distribution pattern of data correlation with network hierarchy. A final line is added regarding this context in the corresponding subsection.

**Query 3:** I suggest the authors should consider revisiting the article's organization to avoid redundancies and provide a cohesive and easy-to-follow narrative.

**Response:** We make a great effort to identify redundancies and remove them from the manuscript to provide a main theme-focused analysis. Thus, we give a more coherent study than before by accepting the suggestion of the reviewer.

**Reviewer 3:**

**Query 1:** Analyzing the Bangladesh stock market is meaningful for the local economy. However, from a global perspective, the importance of the Bangladesh stock market is relatively low compared to countries such as the United States, China, and Japan, for example. Therefore, the authors need to convince people of the implications of analyzing the Bangladesh stock market for financial markets around the world.

**Response:** The stock market in Bangladesh has experienced significant growth in recent years, establishing itself as a prominent participant in the South Asian region. CEIC Data reports that the market capitalization of the Bangladesh stock market reached $41.241 billion in 2023, which is approximately 10.2% of the country's GDP [3]. The market has experienced a rise in foreign investors, with the count of foreign portfolio investors (FPIs) surging from 200 in 2010 to more than 1,000 in 2022 [4]. The market has been garnering the attention of international investors and exhibiting strong performance in recent years. The Bangladesh Securities and Exchange Commission has implemented measures to enhance the transparency and governance of the market, resulting in increased investor interest. We also added the information in the manuscript.

**Query 2:** Why was the MST methodology used? What can we gain from analyzing the hierarchy of a network?

**Response:** By analyzing the MST of a network, we can gain insights into the structure of the network and the relationships between its nodes. The Minimum Spanning Tree (MST) can expose the hierarchical arrangement of a network and it is utilized for identifying the hierarchical structure of financial market sectors. By emphasizing only the most essential connections, MST yields a more hierarchical topological structure and largely eliminates noisy edges. Through a network hierarchy analysis, it is possible to discern the most significant stocks or companies within the network and gain insight into their interrelationships [5, 6, 7].

**Query 3:** Why was normalized log-return used in equation (1)?

**Response:** We corrected the equation (1) in the manuscript. We removed the normalization term from the equation.

**Query 4:** Explain equation (2) in more detail.

**Response:** Equation (2) is corrected with added details in the manuscript.

**Query 5:** The format of the pictures does not match the journal, and the clarity is poor.

**Response:** Pictures are reconstructed by following the journal requirements.

**Query 6:** “which is useful in forecasting any upcoming big crisis” => Is it possible to predict risks? If possible, further explanation and analysis is needed to determine how possible this is.

**Response:** Although it is extremely difficult to predict financial crises in advance, we may suggest that our method may be useful as an indicator because we have observed that, hierarchy values decline drastically before the financial crises we explored.

**Query 7:** "We find that the network hub and central nodes with a maximum degree and BC measure are mainly financial companies, more specifically the insurance companies, i.e., they play important roles in the Bangladeshi market." => What is the reason for these results? Or what do you think?

**Response:** The insurance sector plays a vital role in the Bangladeshi market. According to PwC [8], the insurance sector has the potential to make a positive contribution to the overall standard of living of the country's populace. The sector can help reduce the burden on the overall spending on healthcare. The insurance sector can also help manage risks in an economy at a micro level, safeguarding households and companies from a myriad of risks [8].

**Query 8:** “We need analyzes on other emerging markets like Egypt, Thailand, Indonesia, etc., which will be left for further investigation.” => Please cite related existing literature.

**Response:** Related existing literature of such countries are added in the manuscript.

**Query 9:** “Which can be important to the respective authorities, investors, and policymakers for risk management and portfolio investment.” => Please provide more specific implications.

**Response:** we analysed the time series of DSE data with the evolution of time. We identify the financial events and also influential companies. Our technique might also predict financial market states. In this way investors can decide when they invest to which company at which time. Similarly, policymaker also understand the market behaviour so that they can apply rules to stabilize the market. For example, during the Russia-Ukraine war, the floor price is imposed to resist market fall in Bangladesh.

**References:**

1. <https://www.midwaybd.com/blog/dse-floor-price>
2. <https://www.tbsnews.net/economy/stocks/floor-squeezes-dse-turnover-40-2023-765974>
3. <https://www.ceicdata.com/en/indicator/bangladesh/market-capitalization>
4. <https://www.thedailystar.net/business/economy/stock/news/capital-market-looking-back-looking-forward-3239116>
5. Yu, M., Hillebrand, A., Tewarie, P., Meier, J., van Dijk, B., Van Mieghem, P., & Stam, C. J. (2015). Hierarchical clustering in minimum spanning trees. *Chaos: An Interdisciplinary Journal of Nonlinear Science*, *25*(2).
6. Liu, Y., Lang, B., & Quan, F. (2023). MST-HGCN: a minimum spanning tree hyperbolic graph convolutional network. Applied Intelligence, 53(11), 14515-14526.
7. Sun, M., Zhang, H., Han, D., & Gao, A. (2014). A hierarchical-network-model based analysis of the market characteristics of China's photovoltaic enterprises. Journal of Renewable and Sustainable Energy, 6(4).
8. https://www.pwc.com/bd/en/assets/pdfs/research-insights/2019/potential-for-growth.pdf
