# Supplementary material for: Change in hierarchy of the financial networks: A study on firms of an emerging market in Bangladesh
Source: PLoS One. 2024 May 31;19(5):e0301725. doi: 10.1371/journal.pone.0301725 (PMC11142525; doi:10.1371/journal.pone.0301725)
Supplement: Attachment — Submitted filename: Response to Reviewers.docx [file pone.0301725.s004.docx]

**Rebuttal letter**

PONE-D-23-35741R1

Change in hierarchy of the financial networks: A study on firms of an emerging market in Bangladesh

PLOS ONE

8 March 2024

We thank the reviewers for their valuable comments on our revised manuscript. Below is our response to each point raised by the reviewers. We hope that we satisfyingly addressed them and that the manuscript will be now suited for publication.

We edit our manuscript according to the requisitions of the editor and reviewers. The modifications are inserted in red font in the “Revised Manuscript with Track Changes” file.

Sincerely,

On behalf of all authors,

Dr. Ashadun Nobi

**Responds to Reviewers:**

**Reviewer 2:**

**Suggestion 1:** In the introduction: “The topological...US and China [3] “ ----> “ In particular, the topological...US and China [3]”.

***Response:*** We have modified this line according to your suggestion. Thank you very much for your valuable advice.

**Reviewer 3:**

**Query 1:** Query 7: "We find that the network hub and central nodes with a maximum degree and BC measure are mainly financial companies, more specifically the insurance companies, i.e., they play important roles in the Bangladeshi market." => What is the reason for these results? Or what do you think?

Response: The insurance sector plays a vital role in the Bangladeshi market. According to PwC [8], the insurance sector has the potential to make a positive contribution to the overall standard of living of the country's populace. The sector can help reduce the burden on the overall spending on healthcare. The insurance sector can also help manage risks in an economy at a micro level, safeguarding households and companies from a myriad of risks [8].

Please include the response for the Q7 longer in the manuscript. Other modifications are satisfied.

***Response:*** We have enlarged the response for the Q7 of the previous revision as follows:

“The insurance sector plays a vital role in the Bangladeshi market. For a poor country like Bangladesh, the influence of the insurance company is remarkable since it has been crucial in the lives of mass people and the related aspects those are concerned about. According to PwC [1], the insurance sector has the potential to make a positive contribution to the overall standard of living of the country's populace. The sector can help reduce the burden on the overall spending on healthcare. The insurance sector can also help manage risks in an economy at a micro level, safeguarding households and companies from a myriad of risks [1].

Although the influence of a company depends on various factors such as P/E (Price-Earnings ratio), EPS (Earnings Per Share), ROA (Return on Assets), SZ (Firm’s Size), etc., the average P/E ratio of sample insurance companies in DSE is below 20 (except for 2011) [2], indicating a favorable investment opportunity for small-scale investors. It is also seen that the ROA and EPS of some selected insurance companies have significant positive impacts on market movement. These are a few possible factors why insurance companies are influential in DSE.”

We have included this response in two different sections of the manuscript, in the “Central companies” subsection and “Conclusion” based on its relevancy.

**References:**

1.	Lemmens F. Potential for growth: Transforming Bangladesh’s insurance sector. 2019 [cited 8 Mar 2024]. Available: https://www.pwc.com/bd/en/assets/pdfs/research-insights/2019/potential-for-growth.pdf

2.	Ramij MG, Das A. An Empirical Study on Microeconomic Factors Affecting Stock Price: A Study on Insurance Companies Listed in Dhaka Stock Exchange. Khulna Univ Bus Rev. 2021. doi:10.35649/kubr.2020.15.1.7
